# How are base excision DNA repair pathways deployed *in vivo*?

**DOI:** 10.12688/f1000research.10538.1

**Published:** 2017-03-16

**Authors:** Upasna Thapar, Bruce Demple

**Affiliations:** 1 Department of Pharmacological Sciences, Stony Brook University, School of Medicine, Stony Brook, NY, USA

**Keywords:** Base excision repair, BER, in vivo studies, base excision repair pathways, mammalian cells

## Abstract

Since the discovery of the base excision repair (BER) system for DNA more than 40 years ago, new branches of the pathway have been revealed at the biochemical level by *in vitro* studies. Largely for technical reasons, however, the confirmation of these subpathways *in vivo* has been elusive. We review methods that have been used to explore BER in mammalian cells, indicate where there are important knowledge gaps to fill, and suggest a way to address them.

## Introduction

Cellular DNA is continuously damaged by hydrolytic reactions and metabolic by-products such as free radicals, which results in a wide array of base damages as well as various forms of abasic (AP) residues and deoxyribose fragments ^1^. Damage from environmental agents adds to this burden. Much of the endogenous damage, including most oxidant- or alkylation-induced base lesions, is corrected by the base excision DNA repair (BER) system. The pathways have been summarized in many recent reviews ^2, 3^. Briefly, a set of DNA glycosylases excises the base lesions to generate AP sites that, along with those generated by spontaneous purine loss, are funneled into the central pathways of BER when they are cleaved by an AP endonuclease. In mammalian cells, the main such enzyme is Ape1, which remains at the cleaved site to recruit the next enzyme in the pathway, DNA polymerase β (Polβ). Polβ is quite an inefficient polymerase that may insert only a single nucleotide before completing excision of the residual AP residue to permit DNA ligation. The net result is so-called “short-patch” BER, or more correctly a single-nucleotide pathway (SN-BER). Other variations of SN-BER have been identified: some DNA glycosylases harbor an AP lyase activity that can yield an unsaturated AP residue on the 3’ terminus at the resulting strand break. Excision of the 3’ residue by Ape1 enables Polβ to fill the gap, leading to DNA ligation. Still another reported variation is Ape1 independent ^4, 5^, with the dual lyase activities of NEIL1 or NEIL2 (which are also DNA glycosylases) removing the AP residue to yield a 3’-phosphate. The phosphatase activity of polynucleotide kinase can generate a 3’-OH with the remaining repair steps as above to yield SN-BER.

**Figure 1.**
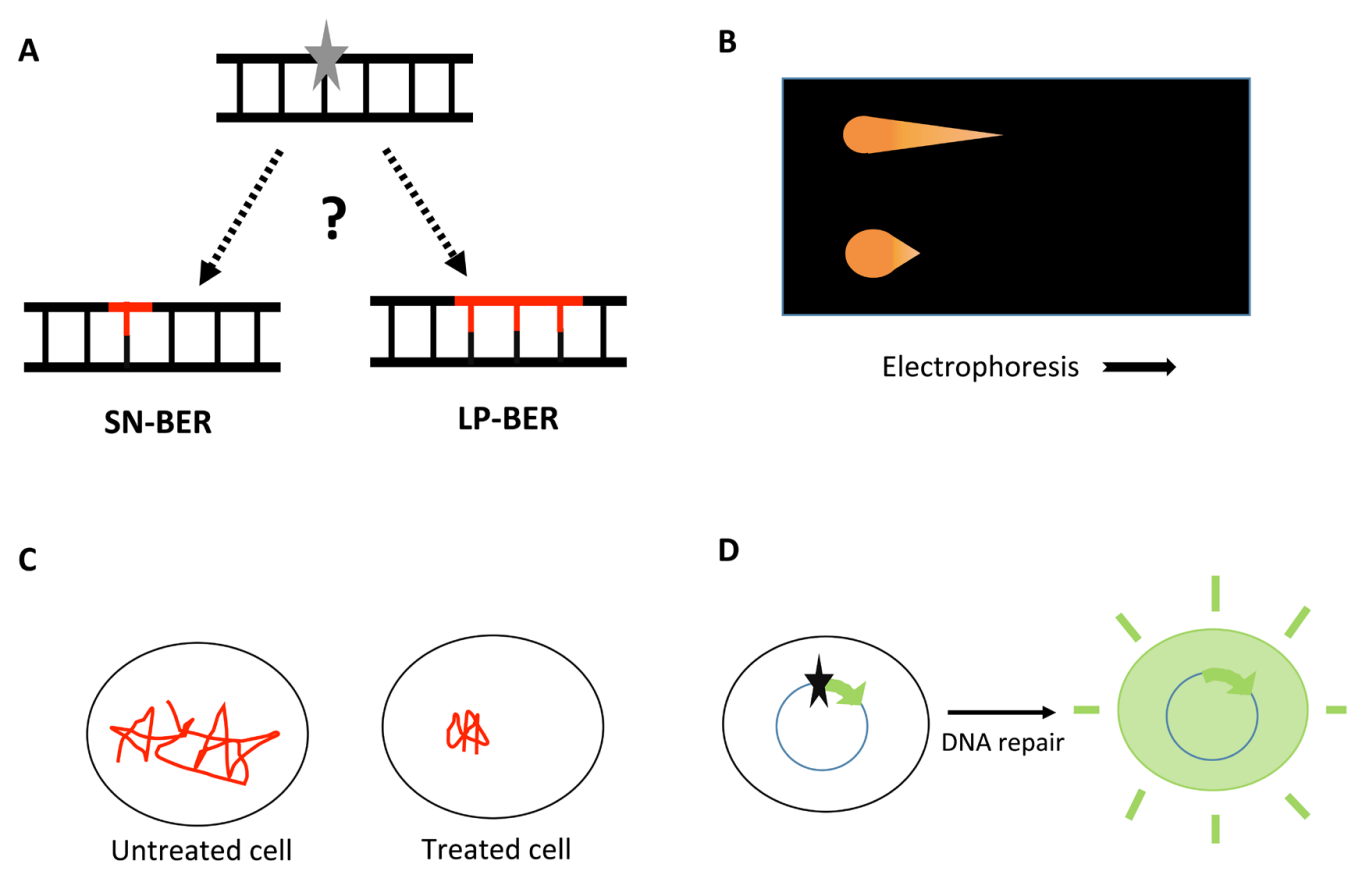
BER products and methods to detect repair *in vivo*. **A**. The products of base excision DNA repair (BER) pathways. Left, single-nucleotide BER (SN-BER), which replaces only the lesion (grey star) itself. Right, “long-patch” BER (LP-BER), which replaces the lesion plus at least the nucleotide 3’ to it to generate repair patches of 2–10 nucleotides. The question mark indicates the uncertainty about the relative contribution of SN-BER and LP-BER *in vivo*. **B**. Schematic of the comet assay, with electrophoresis moving more DNA out of the cell with damage-related strand breaks (upper) than out of the cell with an intact genome (lower). **C**. Schematic of single-molecule tracking in *Escherichia coli*. A DNA repair protein is very mobile in the absence of induced damage. DNA-damaging treatment results in longer residence times at the lesion sites. The total observation time is <1 second. **D**. Host-cell reactivation (HCR) assay restoring gene expression to result in a detectable product (e.g. fluorescence). The star indicates a DNA lesion.

About 20 years ago, *in vitro* studies demonstrated a “long-patch” pathway (LP-BER) ^6– 8^. The key features of LP-BER are the repair DNA synthesis of a more extended segment, 2–10 nucleotides typically ( Figure 1A), and may involve additional DNA polymerases. The extended synthesis prevents the excision of the AP residue by Polβ while generating a displaced oligonucleotide “flap” that requires enzymes such as the FEN1 nuclease to remove it. Ligation then completes the repair. Even for lesions such as uracil in DNA, which would seem to be handled effectively by SN-BER, a substantial fraction of the repair can occur through this pathway in cell-free extracts ^9^. Chemical reduction of an AP site, which prevents its removal by Polβ, caused all the repair to go by LP-BER, and it was speculated that some unknown modified AP lesions might require the pathway ^7^. At least one naturally occurring DNA lesion, 2-deoxyribonolactone, which is generated by many oxidants including intracellular free radicals, can be handled only by LP-BER ^10, 11^. But it is worth noting that virtually all the mechanistic details of BER that we know are the result of *in vitro* studies.

## *In vivo* assays to study BER

A few approaches have been employed to investigate BER *in vivo*. These mainly include cell biology and transfection-based methods such as the comet assay, live cell imaging, and the host-cell reactivation assay.

The comet assay, first used by Ostling and Johanson ^12^ and further developed by Singh *et al.* ^13^, is widely used to follow DNA damage and repair in mammalian cells. Different versions of the assay measure DNA double-strand breaks (the neutral comet assay) or single-strand breaks and alkali-labile sites such as AP lesions (the alkaline comet assay). After treatment and incubation, the cells are cast in low-melting agarose and lysed with mild detergent in a high-salt buffer. The gentle lysis permeabilizes the cells and results in nucleoids, large structures with supercoiled DNA loops attached to nuclear matrix ^14^. Breaks in the DNA loosen these structures, with denaturation at alkaline pH causing further loosening. When the gel-embedded cells are subjected to an electrical field, very little DNA moves out into the gel from cells not treated with DNA-damaging agents. The looser structures in damaged genomes allow more DNA to be electrophoresed out of the cells, and it travels further, leading to the formation of the eponymous “comets” that are visualized using simple fluorescent stains ( Figure 1B). The intensity and length of the comet tail relative to the DNA retained in the nucleus reflects DNA breakage, and the reduction of the comet signal indicates repair ^15^. Modifications of the comet assay to detect some DNA lesions more specifically include the use of DNA repair enzymes, for example *Escherichia coli* endonuclease III (for oxidized pyrimidines) and formamidopyrimidine-DNA glycosylase (for oxidized purines). However, each of those enzymes acts on several base lesions, and both cleave AP sites by the lyase mechanism. As a somewhat inverse application, an *in vitro* comet assay involves incubating DNA nucleoids that contain known amounts of damage with cell-free extracts to measure the repair capacity of cells providing the extracts ^16, 17^. That approach is subject to similar limitations of specificity as the use of enzyme reagents, although the results should relate to the overall cellular BER capacity. Further general limitations of the comet assay to measure BER are that no treatment agent generates only a single DNA lesion, that some level of BER lesions already exists, even in non-treated cells, and that the range of the assay is restricted ^18^.

Another approach to assess DNA repair *in vivo* is fluorescence microscopy, notably techniques such as fluorescence recovery after photobleaching and single-molecule tracking via super-resolution microscopy ^19^. The dynamics of FEN1 at sites of DNA damage following laser irradiation have been investigated using the photobleaching approach ^20^. Quantification can be difficult in studies such as this, as the numbers of lesions and protein molecules per focus are unknown. This problem can be resolved using super-resolution microscopy, which allows tracking of individual molecules in real time inside cells ^21^. Uphoff *et al*. ^22^ employed a combination of single-molecule tracking and photoactivated localization ( Figure 1C) to evaluate the DNA synthesis and ligation steps of BER in bacteria. Fluorescent-tagged DNA polymerase I and DNA ligase were used to measure the reaction rates, spatial distribution, and diffusion characteristics of the proteins in methyl methanesulfonate-damaged and in undamaged cells. These methods can provide insight into the kinetics of specific proteins, overall repair rates, and the organization of repair processes *in vivo.* Similar to the comet assay, which also allows us to assess the repair capacity of individual cells, the single-cell imaging approach enables resolution of the molecular heterogeneity in DNA repair among cells ^19, 23^. However, one must be cognizant of the fact that the measurements are done under experimental conditions which are different from actual physiological settings. Moreover, it is important to verify that the florescent-tagged proteins behave similarly to the endogenous proteins ^19, 24^.

The host-cell reactivation (HCR) assay is another method to monitor cellular repair activity, and it can be employed using viruses or transfected plasmid molecules. The phenomenon was first used to explain the survival of a UV-irradiated bacteriophage in host cells treated with UV and helped lay the groundwork for the discovery of nucleotide excision repair ^25^. Later, it was adapted to study the repair capacity of cells by transfecting them with plasmids ^26– 28^. Instead of survival, the plasmid transfection assay reports the reactivation of expression of a reporter gene (e.g. luciferase) that is blocked by DNA damage and restored by repair dependent on the cellular machinery ( Figure 1D). Depending on the type of damage introduced in the DNA, specific repair systems can be probed. This approach has been extensively used to study the repair of UV-induced damage via nucleotide excision repair in mammalian cells ^27^. The HCR assay has also been used to study the reactivation of viruses treated with monofunctional methylating agents ^29, 30^. Plasmid DNA treated with oxidizing agents such as methylene blue plus visible light has been employed to study the BER capacity of eukaryotic cells ^31^. The approach is not well suited to provide any mechanistic details on how the repair processes in the cell take place, although it can be coupled with known genetic deficiencies to test some models. Indeed, a multiplexing approach has been developed to assess multiple DNA repair pathways simultaneously by using plasmids encoding fluorescent proteins with different excitation and emission maxima, which also allows the use of flow cytometry for the quantification ^32^.

Sattler and co-workers utilized HCR to demonstrate LP-BER *in vivo* ^33^. The indirect approach developed by them was based on evaluating the expression of a reporter gene encoding a fluorescent protein following the repair of a single base lesion on the transcribed strand. Mismatches were also inserted to produce a stop codon read from the transcribed strand and placed at various positions 3’ to the lesion. With this experimental design, repair synthesis beyond the lesion would also correct the stop codon mismatch downstream, indicative of LP-BER. While interesting, this approach has several shortcomings, including, most importantly, the presence of the nearby mismatch. In addition to producing distortion of the DNA structure near the lesion, the mismatches would likely impair ligation during SN-BER, with the effect greatest for the mismatches closest to the lesion. The method did not allow direct measurement of SN-BER. While the experiments were done in a human tumor line deficient in the MLH1 protein, residual mismatch binding or repair, or other repair processes, may well interfere with the interpretation.

All the transfection-based assays suffer from a common shortcoming: they do not account for the effects of packaging DNA in the nucleus into chromatin. DNA lesions in a nucleosome are, in general, processed more slowly than they are in the corresponding free DNA, sometimes by a large difference ^34– 36^. Controlling nucleosome positioning in cells is feasible in some cases, but the fundamental problem of producing a unique DNA lesion for BER, at a known position, would remain a challenge. In principle, it would also be possible to construct molecules for HCR with assembled nucleosomes and including positions from which nucleosomes are excluded. It would be important to confirm that the nucleosomes remain in position after being transfected into cells.

## Need for other *in vivo* assays

BER has been extensively studied *in vitro*. It is clear, though, that there is a dearth of effective techniques and assays enabling the evaluation of the two BER subpathways *in vivo*. The assays discussed above can provide information on the general cellular repair capacity and some kinetics of BER as well as the basis of genetic tests of the roles for specific proteins *in vivo*. However, none of these approaches can address the basic question of what the relative contribution of SN-BER and LP-BER is inside cells. As noted earlier, most *in vitro* studies show SN-BER to be the predominant pathway for some lesions ^37– 39^. Rather unexpectedly, Mosbaugh’s group found LP-BER to be the preferred pathway for uracil repair in mouse fibroblast extracts ^9^. We also found that LP-BER predominated in the repair of even a normal AP site in mouse fibroblast extracts ^10^. But the proportion can vary depending on the type of cell extract and other conditions, so it is hard to know what the *in vivo* distribution is. Although it seems very likely that LP-BER does occur *in vivo*, its existence in cells needs to be verified. To address these issues and provide deeper insights into the mechanistic roles of individual DNA repair proteins, improved *in vivo* assays for BER need to be developed.

It has not been possible to follow the repair of an individual base lesion in genomic DNA for some of the technical reasons already discussed. A next-best approach would be to transfect cells with a plasmid DNA molecule containing a defined single lesion for BER. In other words, HCR but with a twist to allow analysis that would unambiguously distinguish the SN- and LP-BER products generated in cells. We are trying to develop such a method based on mass labeling. This approach depends on designing a plasmid substrate with a site-specific BER lesion, accompanied by nucleotides labeled with stable “heavy” isotopes at various positions around the site. These plasmids will be transfected into cells under conditions not allowing plasmid replication, with an incubation to permit repair and recovery for analysis. Oligonucleotides isolated from the repaired DNA are resolvable by mass spectrometry, depending on the number of heavy nucleotides replaced by normal-mass ones during the processing *in vivo*. Although the lesion nucleotide itself would not be mass labeled, SN-BER would be observed as the loss of sensitivity to lesion-specific enzymes (e.g. uracil-DNA glycosylase) in the recovered DNA molecules. In this way, one could directly measure the amounts of SN- and LP-BER, including a direct assessment of the repair patch size. The approach is not without technical challenges, but it has the potential to address some of the questions we have raised by being applied to a number of well-defined lesions and in various cell backgrounds.

## References

[1] Lindahl T: Instability and decay of the primary structure of DNA. *Nature.* 1993;362(6422):709–15. 10.1038/362709a0 8469282

[2] Schermerhorn KM Delaney S: A chemical and kinetic perspective on base excision repair of DNA. *Acc Chem Res.* 2014;47(4):1238–46. 10.1021/ar400275a 24646203 PMC3993943

[3] Dianov GL Hübscher U: Mammalian base excision repair: the forgotten archangel. *Nucleic Acids Res.* 2013;41(6):3483–90. 10.1093/nar/gkt076 23408852 PMC3616742

[4] Wiederhold L Leppard JB Kedar P: AP endonuclease-independent DNA base excision repair in human cells. *Mol Cell.* 2004;15(2):209–20. 10.1016/j.molcel.2004.06.003 15260972

[5] Das A Wiederhold L Leppard JB: NEIL2-initiated, APE-independent repair of oxidized bases in DNA: Evidence for a repair complex in human cells. *DNA Repair (Amst).* 2006;5(12):1439–48. 10.1016/j.dnarep.2006.07.003 16982218 PMC2805168

[6] Frosina G Fortini P Rossi O: Two pathways for base excision repair in mammalian cells. *J Biol Chem.* 1996;271(16):9573–8. 10.1074/jbc.271.16.9573 8621631

[7] Klungland A Lindahl T: Second pathway for completion of human DNA base excision-repair: reconstitution with purified proteins and requirement for DNase IV (FEN1). *EMBO J.* 1997;16(11):3341–8. 10.1093/emboj/16.11.3341 9214649 PMC1169950

[8] Matsumoto Y Kim K Bogenhagen DF: Proliferating cell nuclear antigen-dependent abasic site repair in *Xenopus laevis* oocytes: an alternative pathway of base excision DNA repair. *Mol Cell Biol.* 1994;14(9):6187–97. 10.1128/MCB.14.9.6187 7915006 PMC359146

[9] Bennett SE Sung JS Mosbaugh DW: Fidelity of uracil-initiated base excision DNA repair in DNA polymerase beta-proficient and -deficient mouse embryonic fibroblast cell extracts. *J Biol Chem.* 2001;276(45):42588–600. 10.1074/jbc.M106212200 11551933

[10] Sung J DeMott MS Demple B: Long-patch base excision DNA repair of 2-deoxyribonolactone prevents the formation of DNA-protein cross-links with DNA polymerase beta. *J Biol Chem.* 2005;280(47):39095–103. 10.1074/jbc.M506480200 16188889

[11] Liu P Qian L Sung JS: Removal of oxidative DNA damage via FEN1-dependent long-patch base excision repair in human cell mitochondria. *Mol Cell Biol.* 2008;28(16):4975–87. 10.1128/MCB.00457-08 18541666 PMC2519700

[12] Ostling O Johanson KJ: Microelectrophoretic study of radiation-induced DNA damages in individual mammalian cells. *Biochem Biophys Res Commun.* 1984;123(1):291–298. 10.1016/0006-291X(84)90411-X 6477583

[13] Singh NP McCoy MT Tice RR: A simple technique for quantitation of low levels of DNA damage in individual cells. *Exp Cell Res.* 1988;175(1):184–191. 10.1016/0014-4827(88)90265-0 3345800

[14] Cook PR Brazell IA Jost E: Characterization of nuclear structures containing superhelical DNA. *J Cell Sci.* 1976;22(2):303–24. 1002771 10.1242/jcs.22.2.303

[15] Azqueta A Collins AR: The essential comet assay: a comprehensive guide to measuring DNA damage and repair. *Arch Toxicol.* 2013;87(6):949–68. 10.1007/s00204-013-1070-0 23685795

[16] Collins AR Dusinská M Horváthová E: Inter-individual differences in repair of DNA base oxidation, measured *in vitro* with the comet assay. *Mutagenesis.* 2001;16(4):297–301. 10.1093/mutage/16.4.297 11420396

[17] Azqueta A Slyskova J Langie SA: Comet assay to measure DNA repair: approach and applications. *Front Genet.* 2014;5:288. 10.3389/fgene.2014.00288 25202323 PMC4142706

[18] Collins AR: Investigating oxidative DNA damage and its repair using the comet assay. *Mutat Res.* 2009;681(1):24–32. 10.1016/j.mrrev.2007.10.002 18054270

[19] Uphoff S Kapanidis AN: Studying the organization of DNA repair by single-cell and single-molecule imaging. *DNA Repair (Amst).* 2014;20:32–40. 10.1016/j.dnarep.2014.02.015 24629485 PMC4119245

[20] Kleppa L Mari PO Larsen E: Kinetics of endogenous mouse FEN1 in base excision repair. *Nucleic Acids Res.* 2012;40(18):9044–59. 10.1093/nar/gks673 22810208 PMC3467068

[21] Betzig E Patterson GH Sougrat R: Imaging intracellular fluorescent proteins at nanometer resolution. *Science.* 2006;313(5793):1642–5. 10.1126/science.1127344 16902090

[22] Uphoff S Reyes-Lamothe R Garza de Leon F: Single-molecule DNA repair in live bacteria. *Proc Natl Acad Sci U S A.* 2013;110(20):8063–8. 10.1073/pnas.1301804110 23630273 PMC3657774

[23] Olive PL Banáth JP: The comet assay: a method to measure DNA damage in individual cells. *Nat Protoc.* 2006;1(1):23–9. 10.1038/nprot.2006.5 17406208

[24] Karanam K Loewer A Lahav G: Dynamics of the DNA damage response: insights from live-cell imaging. *Brief Funct Genomics.* 2013;12(2):109–17. 10.1093/bfgp/els059 23292635 PMC3609438

[25] Howard-Flanders P: DNA repair. *Annu Rev Biochem.* 1968;37:175–200. 10.1146/annurev.bi.37.070168.001135 4875714

[26] Protić-Sabljić M Kraemer KH: One pyrimidine dimer inactivates expression of a transfected gene in xeroderma pigmentosum cells. *Proc Natl Acad Sci U S A.* 1985;82(19):6622–6. 10.1073/pnas.82.19.6622 2995975 PMC391262

[27] Johnson JM Latimer JJ: Analysis of DNA repair using transfection-based host cell reactivation. *Methods Mol Biol.* 2005;291:321–35. 10.1385/1-59259-840-4:321 15502233 PMC4860737

[28] Chu G Berg P: DNA cross-linked by cisplatin: a new probe for the DNA repair defect in xeroderma pigmentosum. *Mol Biol Med.* 1987;4(5):277–90. 3695939

[29] Day RS 3rd Ziolkowski CH: Human brain tumour cell strains with deficient host-cell reactivation of *N*-methyl- *N'*-nitro- *N*-nitrosoguanidine-damaged adenovirus 5. *Nature.* 1979;279(5716):797–9. 10.1038/279797a0 450131

[30] Maynard K Parsons PG Cerny T: Relationships among cell survival, O6-alkylguanine-DNA alkyltransferase activity, and reactivation of methylated adenovirus 5 and herpes simplex virus type 1 in human melanoma cell lines. *Cancer Res.* 1989;49(17):4813–7. 2547518

[31] Kassam SN Rainbow AJ: UV-inducible base excision repair of oxidative damaged DNA in human cells. *Mutagenesis.* 2009;24(1):75–83. 10.1093/mutage/gen054 18836099

[32] Nagel ZD Margulies CM Chaim IA: Multiplexed DNA repair assays for multiple lesions and multiple doses via transcription inhibition and transcriptional mutagenesis. *Proc Natl Acad Sci U S A.* 2014;111(18):E1823–32. 10.1073/pnas.1401182111 24757057 PMC4020053

[33] Sattler U Frit P Salles B: Long-patch DNA repair synthesis during base excision repair in mammalian cells. *EMBO Rep.* 2003;4(4):363–7. 10.1038/sj.embor.embor796 12671676 PMC1319152

[34] Nilsen H Lindahl T Verreault A: DNA base excision repair of uracil residues in reconstituted nucleosome core particles. *EMBO J.* 2002;21(21):5943–52. 10.1093/emboj/cdf581 12411511 PMC131078

[35] Cole HA Tabor-Godwin JM Hayes JJ: Uracil DNA glycosylase activity on nucleosomal DNA depends on rotational orientation of targets. *J Biol Chem.* 2010;285(4):2876–85. 10.1074/jbc.M109.073544 19933279 PMC2807341

[36] Rodriguez Y Smerdon MJ: The structural location of DNA lesions in nucleosome core particles determines accessibility by base excision repair enzymes. *J Biol Chem.* 2013;288(19):13863–75. 10.1074/jbc.M112.441444 23543741 PMC3650422

[37] Dianov G Price A Lindahl T: Generation of single-nucleotide repair patches following excision of uracil residues from DNA. *Mol Cell Biol.* 1992;12(4):1605–12. 10.1128/MCB.12.4.1605 1549115 PMC369603

[38] Kubota Y Nash RA Klungland A: Reconstitution of DNA base excision-repair with purified human proteins: interaction between DNA polymerase beta and the XRCC1 protein. *EMBO J.* 1996;15(23):6662–70. 8978692 PMC452490

[39] Fortini P Pascucci B Parlanti E: 8-Oxoguanine DNA damage: at the crossroad of alternative repair pathways. *Mutat Res.* 2003;531(1–2):127–39. 10.1016/j.mrfmmm.2003.07.004 14637250

